# The effectiveness of interventions to reduce the household economic burden of illness and injury: a systematic review

**DOI:** 10.2471/BLT.14.139287

**Published:** 2014-11-18

**Authors:** Beverley M Essue, Merel Kimman, Nina Svenstrup, Katharina Lindevig Kjoege, Tracey Lea Laba, Maree L Hackett, Stephen Jan

**Affiliations:** a The George Institute for Global Health, PO Box M201, Missenden Road, Camperdown, NSW 2050, Australia.

## Abstract

**Objective:**

To determine the nature, scope and effectiveness of interventions to reduce the household economic burden of illness or injury.

**Methods:**

We systematically reviewed reports published on or before 31 January 2014 that we found in the CENTRAL, CINAHL, Econlit, Embase, MEDLINE, PreMEDLINE and PsycINFO databases. We extracted data from prospective controlled trials and assessed the risk of bias. We narratively synthesized evidence.

**Findings:**

Nine of the 4330 studies checked met our inclusion criteria – seven had evaluated changes to existing health-insurance programmes and two had evaluated different modes of delivering information. The only interventions found to reduce out-of-pocket expenditure significantly were those that eliminated or substantially reduced co-payments for a given patient population. However, the reductions only represented marginal changes in the total expenditures of patients. We found no studies that had been effective in addressing broader household economic impacts – such as catastrophic health expenditure – in the disease populations investigated.

**Conclusion:**

In general, interventions designed to reduce the complex household economic burden of illness and injury appear to have had little impact on household economies. We only found a few relevant studies using rigorous study designs that were conducted in defined patient populations. The studies were limited in the range of interventions tested and they evaluated only a narrow range of household economic outcomes. There is a need for method development to advance the measurement of the household economic consequences of illness and injury and facilitate the development of innovative interventions to supplement the strategies based on health insurance.

## Résumé

**Objectif:**

Déterminer la nature, la portée et l'efficacité des interventions visant à réduire la charge économique des maladies ou des blessures sur les ménages.

**Méthodes:**

Nous avons systématiquement passé en revue les rapports publiés avant le ou à la date du 31 janvier 2014, que nous avons trouvés dans les bases de données CENTRAL, CINAHL, Econlit, Embase, MEDLINE, PreMEDLINE et PsycINFO. Nous avons extrait les données à partir d'essais contrôlés prospectifs et évalué le risque de biais. Nous avons fait la synthèse des données de manière narrative.

**Résultats:**

Parmi les 4 330 études examinées, 9 d'entre elles ont satisfait nos critères d'inclusion – 7 avaient évalué les changements dans les programmes d'assurance maladie existants et 2 avaient évalué les différents modes de diffusion des informations. Les seules interventions qui réduisaient significativement les dépenses restant à la charge des patients étaient celles qui éliminaient ou diminuaient substantiellement la participation aux frais pour une population de patients donnée. Toutefois, les réductions ne représentaient que des changements marginaux dans l'ensemble des dépenses des patients. Nous n'avons trouvé aucune étude qui n'ait été efficace dans le traitement des impacts économiques plus larges sur les ménages – comme les dépenses catastrophiques de santé – dans les populations de malades étudiées.

**Conclusion:**

En général, les interventions visant à réduire la charge économique et complexe des maladies et des blessures sur les ménages semblent n'avoir que peu d'effet sur l'économie des ménages. Nous n'avons trouvé qu'un petit nombre d'études pertinentes qui utilisaient des modèles d'étude rigoureux et qui ont été menées sur des populations de patients définies. Les études ont été limitées dans la gamme des interventions testées et elles n'ont évalué qu'une gamme restreinte de résultats économiques sur les ménages. Il est nécessaire de développer des méthodes pour améliorer la quantification des conséquences économiques des maladies et des blessures sur les ménages et pour faciliter le développement d'interventions innovantes afin de compléter les stratégies reposant sur l'assurance maladie.

## Resumen

**Objetivo:**

Determinar la naturaleza, el alcance y la eficacia de las intervenciones para reducir la carga económica familiar de enfermedades o lesiones.

**Métodos:**

Se revisaron sistemáticamente los informes publicados hasta el 31 de enero de 2014 (incluido) procedentes de las bases de datos CENTRAL, CINAHL, Econlit, Embase, MEDLINE, PreMEDLINE y PsycINFO. Se extrajeron los datos de ensayos controlados prospectivos y se evaluó el riesgo de sesgo. Posteriormente, se sintetizaron narrativamente las pruebas.

**Resultados:**

Nueve de los 4330 estudios examinados cumplieron con los criterios de inclusión: siete habían evaluado los cambios en los programas de seguros de salud existentes, mientras que los otros dos habían evaluado modos diferentes de transmisión de la información. Se halló que las intervenciones que eliminaban o reducían sustancialmente los copagos para una población de pacientes concreta eran las únicas que reducían el desembolso directo de forma significativa. Sin embargo, las reducciones solo representaban cambios marginales en el gasto total de los pacientes. No se encontraron estudios que hubieran abordado con eficacia los efectos mayores en la economía familiar, como los gastos catastróficos por motivos de salud en las poblaciones de enfermedad investigadas.

**Conclusión:**

En general, las intervenciones destinadas a reducir la complejidad de la carga económica familiar de enfermedades y lesiones parecen haber afectado poco a la economía familiar. Se encontraron pocos estudios relevantes con un diseño de estudio riguroso realizados en poblaciones de pacientes definidas. Estos estudios se limitaron a un conjunto de intervenciones probadas y únicamente evaluaban un conjunto reducido de resultados económicos para los hogares. Es necesario elaborar métodos para avanzar en la medición de las consecuencias económicas de enfermedades y lesiones para los hogares, además de facilitar el desarrollo de intervenciones innovadoras a fin de complementar las estrategias basadas en seguros de salud.

## ملخص

**الغرض:**

تحديد طبيعة ونطاق وفعالية التدخلات الرامية إلى تقليل العبء الاقتصادي للمرض أو الإصابة لدى الأسر المعيشية.

**الطريقة:**

قمنا بإجراء مراجعة منهجية للتقارير المنشورة بتاريخ 31 كانون الثاني/ يناير 2014 أو ما بعده التي تم العثور عليها في قواعد بيانات CENTRAL وCINAHL وEconlit وEmbase وMEDLINE وPreMEDLINE وPsycINFO. وقمنا باستخلاص البيانات من التجارب الاستطلاعية التي أجريت في بيئة خاضعة للمراقبة وتقييم مخاطر التحيز. وقمنا باستخلاص البيّنات على نحو سردي.

**النتائج:**

استوفت تسع دراسات من أصل 4330 دراسة تم فحصها معايير الإدراج المحددة من قبلنا - قامت سبع منها بتقييم التغيرات في برامج التأمين الصحي الحالية وقامت اثنتان بتقييم الأنماط المختلفة لإيتاء المعلومات. وكانت التدخلات الوحيدة التي تبين أنها تقلل النفقات التي ينفقها الفرد من جيبه الخاص بشكل كبير تلك التي ألغت المدفوعات المشتركة أو خفضتها بشكل كبير لمرضى معينين. ومع ذلك، لم تمثل الانخفاضات سوى تغيرات هامشية في إجمالي نفقات المرضى. ولم نجد دراسات فعالة في معالجة الآثار الاقتصادية الأعم على الأسر المعيشية - مثل الإنفاق الكارثي في مجال الصحة - في المرضى الذين تم تحريهم.

**الاستنتاج:**

بشكل عام، يبدو أن التدخلات المصممة لتقليل العبء الاقتصادي المعقد للمرض والإصابة لدى الأسر المعيشية كانت ذات أثر ضئيل على اقتصاديات الأسر المعيشية. ولم نجد سوى بضع دراسات ذات صلة تستخدم تصاميم الدراسة الصارمة التي تم إجراؤها على مرضى محددين. وكانت الدراسات محدودة في نطاق التدخلات التي تم اختبارها ولم تقم إلا بتقييم نطاق ضيق من الحصائل الاقتصادية لدى الأسر المعيشية. وثمة حاجة لوضع أسلوب لإحراز تقدم في قياس الآثار الاقتصادية للمرض والإصابة لدى الأسر المعيشية وتسهيل وضع تدخلات ابتكارية لاستكمال الاستراتيجيات المستندة على التأمين الصحي.

## 摘要

**目的:**

确定减少疾病或损伤的家庭经济负担的干预措施的性质、范围和有效性。

**方法:**

我们系统地回顾了在CENTRAL、CINAHL、Econlit、Embase、MEDLINE、PreMEDLINE和PsycINFO数据库中找到的发表于2014年1月31日或之前的报告。我们提取前瞻性对照试验的数据并评估偏差的风险。我们以叙事形式合成证据。

**结果:**

在检查的4330项研究中有九项满足我们的入选标准，其中七项研究对现有的医疗保险计划的变更做出评估，两项研究对不同信息交付模式进行评估。我们发现的唯一可显著减少自付费用的干预措施是消除或大大减少给定病人群体的共付额。然而，这种减少只代表病人总支出的边际变化。我们没有发现有效地解决所调查人口中更广泛的家庭经济影响（如灾难性卫生支出）的研究。

**结论:**

一般来说，旨在减少疾病和损伤复合家庭经济负担的干预措施似乎对家庭经济影响不大。我们只找到了很少几项使用严格研究设计并在限定患者群体中执行的相关研究。这些研究的范围限于检测和评估狭小范围家庭经济产出的干预措施。需要发展测量疾病和损伤家庭经济后果的方法，并促进发展创新的干预措施以作为基于医疗保险的战略的补充。

## Резюме

**Цель:**

Определить характер, масштабы и эффективность мер по снижению экономического бремени болезней и травм для домохозяйств.

**Методы:**

Проводился систематический обзор отчетов, опубликованных по состоянию на 31 января 2014 года в базах данных CENTRAL, CINAHL, Econlit, Embase, MEDLINE, PREMEDLINE и PsycInfo. Были извлечены данные из проспективных контролируемых исследований и определен риск системной ошибки. Для собранных данных была проведена описательная классификация.

**Результаты:**

Девять из 4330 рассмотренных исследований соответствовали критериям включения в обзор — в семи из них оценивались изменения в существующих программах медицинского страхования, а в двух исследованиях оценивались различные способы доставки информации. Единственными выявленными мероприятиями, которые приводили к существенному снижению собственных расходов домохозяйств на лечение, были те, которые устраняли или значительно сокращали собственные доплаты для определенного контингента больных. Тем не менее, это снижение собственных доплат составляло лишь незначительную долю в общем объеме расходов пациентов. Не были найдены исследования, которые бы эффективно устраняли экономические воздействия на домохозяйства — такие как катастрофические расходы на здравоохранение — в исследованных группах заболеваний.

**Вывод:**

В целом, выявленные меры, направленные на снижение сложного экономического бремени болезней и травм для домохозяйств, оказывали незначительное влияние на экономику этих домохозяйств. Были обнаружили всего несколько соответствующих исследований, использующих тщательно разработанные схемы, которые были проведены в определенных группах пациентов. Эти исследования касались ограниченного диапазона мероприятий и оценивали лишь узкий диапазон воздействий на экономику домохозяйств. Существует потребность в разработке методов измерения экономических последствий болезней и травм для домохозяйств, как и в содействии разработке инновационных мероприятий в дополнение к стратегиям, основанным на медицинском страховании.

## Introduction

Each year, globally, around 150 million people struggle to meet the costs of accessing and using health care and approximately 100 million people are driven below the poverty line by such costs.^1^ Many people delay or avoid health care because it is – or, at least, is perceived to be – unaffordable.^2^^–^^4^ Most of those who struggle to meet the out-of-pocket costs of health care live in low-income countries that have poorly funded health systems and inadequate measures to ensure the financial protection of households against high health-care expenditure. However, the problem is not limited to such countries. In 2007, for example, 62% of the personal bankruptcies recorded in the United States of America (USA) were attributed to medical debt^5^ and 11% of the individuals found insolvent in Australia cited ill-health or lack of health insurance as the primary reason for their insolvency.^6^ Substantial and unpredictable one-off health-care payments and a steady flow of unbudgeted medical bills can lead many households – particularly those already marginalized by socioeconomic disadvantage – towards catastrophic health-care expenditure.^7^

The economic burden of illness in a household is only partly explained by out-of-pocket expenditure. The full evaluation of such burden requires a multidimensional framework – to move beyond absolute spending to incorporate measures that examine the broader impacts of illness or injury on the household economy – e.g. loss of employment – as well as the affordability of care, a household’s response to an injury or illness and the consequences of those responses for the household.^8^^,^^9^ Most research in this area has been observational and has demonstrated that households will employ several strategies – to deal with unbudgeted costs of medical care and unplanned departures from the workforce – when coping with the onset of an illness or injury, especially in the main income earner. Such coping strategies include drawing on available social resources and networks, cutting back on essential living expenses, drawing on savings, selling assets, borrowing money, entering into formal or informal loan agreements, increasing credit or debt and even moving house.^3^^,^^4^ Although these strategies may help leverage the resources needed to pay for care, they can also have adverse effects on treatment-seeking behaviour and the long-term economic well-being and resilience of the household.^3^^,^^4^^,^^7^

The provision of adequate financial protection – from the costs of seeking and using medical care – is a critical marker of the effectiveness of a health-care system.^10^ The World Health Organization has encouraged its Member States to provide universal health coverage in some form and the United Nations has recently passed a declaration that calls for universal access to health care that does not cause financial hardship.^11^ Such a goal – like other post-2015 development goals aimed at alleviating poverty – is unlikely to be achieved without further development and implementation of national health-insurance schemes. There is considerable evidence, most notably from the RAND Health Insurance Experiments,^12^ that indicates how health insurance can protect the finances of households affected by illness or injury, by restricting individual health-care expenditure. However, although such insurance is one of the most important population-based policy interventions to mitigate the economic burden of injury or illness, it is not sufficient, on its own, to provide full protection from catastrophic health expenditure.^13^^,^^14^ The effectiveness of health insurance in protecting individuals who are intense users of medical care – e.g. those with chronic illness or long-term injuries – has yet to be elucidated. Furthermore, limited coverage of services and high levels of co-payment can often mean that households with health insurance remain at risk of catastrophic health-care expenditures and economic hardship.^14^^,^^15^

Evidence of the effectiveness of simple education and support interventions, in both clinic- and community-based settings, has highlighted the potential value of more targeted and patient-focused strategies in reducing the household economic burden of illness. Interventions that help patients and caregivers to navigate through health and social-welfare support systems^16^^,^^17^ and informal loan and microcredit schemes^18^^–^^20^ have the potential to buffer those with illness and injury against financial hardship. As the evidence of the effectiveness and cost–effectiveness of such interventions becomes more robust, opportunities for the development and scale-up of such interventions need to be explored.

There have been few systematic reviews of interventions to reduce the household economic burden of illness or injury. The reviews that have been conducted have tended to take a population-based approach – e.g. they have examined the impact of health-insurance programmes on entire populations – and have often been based on studies that involved retrospective comparisons of before and after data. Furthermore, they have focused either on specific types of interventions – e.g. programmes for the management of chronic illness^21^ or health-insurance schemes^22^^–^^24^ – or have focused, narrowly, on out-of-pocket payments, as the sole measure of the economic impact of illness.^24^ We decided to conduct a systematic review to try to determine the nature, scope and effectiveness of all interventions that have been designed to reduce the household economic burden of illness or injury.

## Methods

We searched electronic databases, using a predefined search strategy and confining the search to reports published on or before 31 January 2014 (Box 1). The reference lists of retrieved articles were screened to identify additional studies, and investigators known to be carrying out relevant research were contacted for unpublished data. Non-English articles were translated where necessary.

Box 1 **Basic literature search strategy for systematic review of interventions to reduce the household economic burden of ill health**

The following databases were searched: CENTRAL, CINAHL, Econlit, Embase, MEDLINE, PreMEDLINE and PsycINFO

Search terms:

1. “intervention” OR “program” OR “programme” OR “policy” OR “scheme”

2. “catastrophic” AND “finance OR cost OR medical OR expenditure”

3. “finance OR economic” AND “hardship OR strain OR stress OR well-being”

4. “burden” AND “household financial OR household economic”

5. “household” AND “economic impact”

6. “out-of-pocket” AND “cost OR expenditure OR spend OR payment OR catastrophic”

A detailed search strategy for each database is available from the authors.

To be included in our review, a study (i) had to be a prospective controlled trial of one or more interventions – i.e. a randomized or nonrandomized controlled trial, an interrupted time series study with control, or a controlled before-and-after study; (ii) involve a study population with any, chronic or acute, communicable or noncommunicable disease or injury; and (iii) use a study outcome that was a measure of the household economic burden of illness or injury – e.g. out-of-pocket expenditure or level of economic hardship.

Interventions directed at the individual, household or population and delivered in any setting were eligible for inclusion. Studies that were primarily treatment or medical interventions – e.g. cataract surgery or chemotherapy – were excluded even if they included economic measures as additional outcomes.

Two authors carried out the literature search and screened titles and abstracts using a standardized eligibility assessment form based on our inclusion criteria. The full texts of articles of potential interest were reviewed by two authors and a final decision on which studies to include was confirmed by consensus. A third author provided arbitration if consensus was not reached. One author used a predefined form^25^^,^^26^ to extract data from each included study. The data extraction was verified by a second author. Authors of included studies were contacted for any missing information or data. Where possible, effect estimates were calculated as standardized mean differences between the intervention and control groups, with 95% confidence intervals.^27^ Where reported, data on the impact of the interventions on health-service-utilization – e.g. numbers of hospital admissions or medical appointments – and medication adherence were also collected.

The risk of bias in each of the included studies was assessed by one author –using the criteria suggested for Effective Practice and Organisation of Care reviews^28^ – and verified by a second author.

Quantitative analysis of the data was deemed inappropriate because of the heterogeneity in the collected data, designs and settings of the included studies.

## Results

**Fig. 1:**
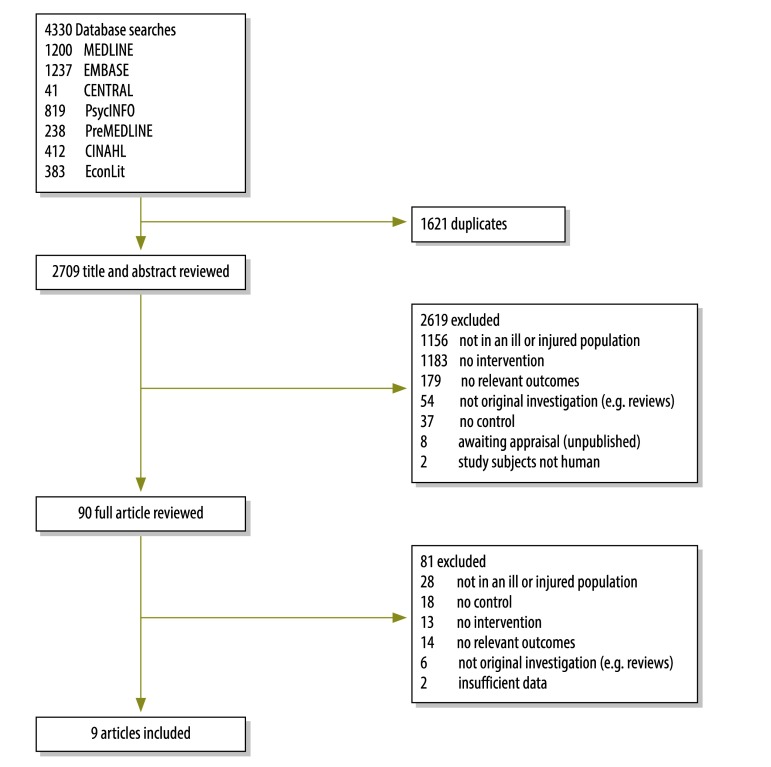
Flowchart for the selection of studies on interventions to reduce the household economic burden of ill health

The initial literature search identified 4330 citations. There were 90 articles of potential interest and, after examination of the full texts, nine articles described studies that met all of our inclusion criteria (Fig. 1). Each of the nine articles – seven conducted in the USA,^29^^–^^35^ one in Finland^36^ and one in China^37^ – described a single study. Most of the included studies had investigated adult urban patients with noncommunicable disease (6/9) and had involved data from more than 1000 participants (7/9; Table 1). Illness and injury inclusion criteria had been assessed using diagnostic codes, the health-service use reported in insurance claims, clinical presentations or self-reporting.

**Table 1:** Characteristics of the included studies on interventions to reduce the household economic burden of ill health

Study	Country	Study design (sample size)	Study objective	Study population	Intervention vs control	Outcomes measured
Jing et al. (2013)^37^	China	CBA (*n* = 2998)	To evaluate the impact of the New Cooperative Medical Schemes’ reimbursement policies for chronic disease	Rural households in which one or more members have self-reported chronic disease	Higher reimbursement for essential drugs – e.g. 80% for diabetes and hypertension medications – and outpatient care – e.g. 25% or 40% – for specified chronic diseases vs usual care	Catastrophic health expenditure
Heikkinen et al. (2011)^36^	Finland	NRCT (*n* = 147)	To evaluate cost of care between two different modes of delivering patient education	Ambulatory orthopaedic surgery patients	Website containing biophysiological, social and financial information plus email contact with nurse vs face-to-face education	Out-of-pocket costs
Barry et al. (2013)^29^	USA	CBA (*n* = 1 907 218)	To evaluate the impact of FEHBP parity policy on out-of-pocket costs	Individuals aged ≤ 21 years with MH/SUD	Parity of benefits for MH/SUD services vs usual care	Share of total costs spent on MH/SUD services and mean out-of-pocket costs for MH/SUD
Busch et al. (2013)^30^	USA	CBA (*n* = 29 615)	To evaluate the impact of FEHBP parity policy on spending and intensity of service use	Enrolees of FEHBP with bipolar disorder, major depression or adjustment disorder	Parity of benefits for MH/SUD services vs usual care	Out-of-pocket costs and health-service utilization
Choudhry et al. (2011)^31^	USA	RCT (*n* = 5855)	To evaluate the effect of providing full prescription drug coverage	Individuals with a principal or secondary diagnosis of acute myocardial infarction	Full prescription drug coverage vs usual coverage	Medication adherence and health-care spending, including out-of-pocket costs
Choudhry et al. (2012)^32^	USA	ITS (*n* = 52 631)	To evaluate the impact of reductions in drug co-payments	Individuals with diabetes or vascular disease	Co-payment elimination for patients with diabetes and reduction for patients on clopidogrel vs usual coverage	Out-of pocket costs, medication use and health-service utilization
Davidoff et al. (2005)^33^	USA	ITS (*n* = 3413)	To evaluate the effects of the expansion of the SCHIP	Children with chronic health conditions	Expansion of eligibility for SCHIP vs usual care	Out-of-pocket spending and health-service use
Goldman et al. (2006)^34^	USA	ITS (*n* = 320 000)	To evaluate the impact of parity in insurance benefits	Enrolees of the FEHBP accessing MH/SUD services	Parity of benefits for MH/SUD services vs usual care	Rate of MH/SUD utilization, out-of-pocket costs and total spending
Van Houtven et al. (2013)^35^	USA	RCT (*n* = 187)	To evaluate the effect of a multicomponent intervention for caregivers of older adults	Caregivers of patients with Alzheimer or Parkinson diseases	Multicomponent training over 24 weeks for caregivers via the ASSIST programme vs social phone contacts while on waiting list	Out-of-pocket costs

ASSIST: Assistance, Support, and Self-health Initiated through Skill Training; CBA: controlled before-and-after study; FEHBP: Federal Employees Health Benefits Program; ITS: interrupted time series; MH/SUD: mental health and substance use disorders; NRCT: nonrandomized controlled trial; RCT: randomized controlled trial; SCHIP: State Children’s Health Insurance Program: USA: United States of America.

Seven of our included studies had evaluated policy interventions that involved health-insurance schemes (Table 2). Of these, three had involved the reduction or elimination of co-payments for disease-specific medications or outpatient care.^31^^,^^32^^,^^37^ Another three studies had evaluated the effectiveness of a similar intervention – that offered parity in service coverage for mental health and substance use disorders – in different subgroups.^29^^,^^30^^,^^34^ One study had investigated the extension of coverage of an existing health-insurance scheme to a new patient population.^33^

**Table 2:** Characteristics of interventions investigated in the included studies on interventions to reduce the household economic burden of ill health

Study	Intervention details	Setting	Target population
Choudhry et al. (2011)^31^	Health-insurance policy – elimination of co-payments for disease-specific drugs	Health-insurance programme	Enrolees
Choudhry et al. (2012)^32^	Health-insurance policy – reduction or elimination of co-payments for disease-specific drugs	Health-insurance programme	Enrolees
Jing et al. (2013)^37^	Health-insurance policy – higher reimbursement for outpatient ambulatory services and drugs	County population	Enrolees, rural
Davidoff et al. (2005)^33^	Health-insurance policy – extended insurance coverage	Health-insurance programme	Enrolees, children
Goldman et al. (2006)^34^	Health-insurance policy – parity of coverage for disease-specific services	Health-insurance programme	Enrolees
Barry et al. (2013)^29^	Health-insurance policy – parity of coverage for disease-specific services	Health-insurance programme	Enrolees, children
Busch et al. (2013)^30^	Health-insurance policy – parity of coverage for MH/SUD services	Health-insurance programme	Enrolees
Heikkinen et al. (2013)^36^	Delivery of information and support using a web-based platform	Health service	Clinic-based population
Van Houtven et al. (2013)^35^	Delivery of information and support using telephone and in-person training	Health service	Clinic-based population, caregivers

MH/SUD: mental health and substance use disorders.

The other two studies trialled different models of delivering patient-focused education and support – e.g. by web- or telephone-based communication or in-person.^35^^,^^36^

Out-of-pocket expenditure had been the primary outcome in six of our included studies – including one post-hoc analysis – and a supplementary outcome in another two (Table 1). The researchers involved in most of the studies had ascertained out-of-pocket expenditures from databases of insurance claims. Household economic burden had also been measured in terms of the likelihood of a household paying any out-of-pocket costs for care, the prevalence of catastrophic health expenditure – i.e. out-of-pocket costs that were greater than 40% of the maximum amount that a household could pay – and the prevalence of cost-related delays in seeking care. None of the studies had evaluated the effectiveness of an intervention in reducing economic hardship.

Six of the studies had also investigated the effectiveness of an intervention on clinical and health-system outcomes, health-service use, adherence to pharmaceuticals, direct costs to private health insurers or the indirect costs to patients and household caregivers in terms of the time spent seeking health care.

**Fig. 2:**
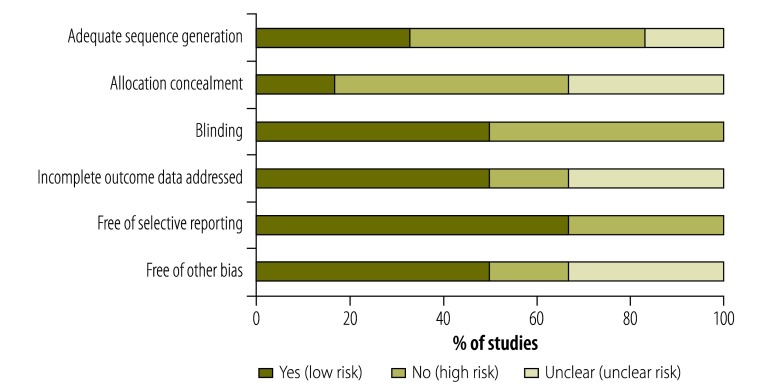
Risk of bias in the randomized and nonrandomized controlled trials and the controlled before-and-after studies on interventions to reduce the household economic burden of ill health

Note: Each domain refers to an area of potential bias that could affect the validity of the six studies. For each domain, a study was categorized as high, low or unclear risk, using the criteria suggested for Effective Practice and Organisation of Care reviews.^28^

There was a high or unclear risk of bias in the randomized and nonrandomized controlled trials and controlled before-and-after studies (Fig. 2; available from: http://www.who.int/bulletin/volumes/93/2/14-139287). In these studies, inadequate allocation-sequence generation and concealment could have resulted in an overestimate of the effects of an intervention on the household economic burden – particularly since absolute out-of-pocket expenditure was often the main outcome and such expenditure was self-reported in three studies.^35^^–^^37^ Attrition bias due to incomplete reporting of outcome data – which may also lead to overestimates of an intervention – was potentially an issue in three studies.^35^^–^^37^ There was also a high risk of reporting bias in two of the studies.^35^^,^^36^

**Fig. 3:**
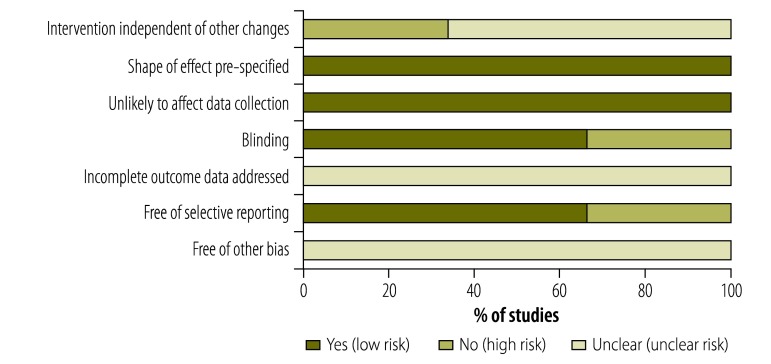
Risk of bias in the interrupted time series studies on interventions to reduce the household economic burden of ill health

Note: Each domain refers to an area of potential bias that could affect the validity of the three studies. For each domain, a study was categorized as high, low or unclear risk, using the criteria suggested for Effective Practice and Organisation of Care reviews.^28^

The data we reviewed from interrupted time series studies (3/9) had a generally low risk of bias (Fig. 3; available from: http://www.who.int/bulletin/volumes/93/2/14-139287). However, in such studies, there is some risk that the intervention effect may not have occurred independently of other changes occurring over time and that the outcome observed may have been influenced by confounding factors. These two issues may have resulted in an overestimate of the effect of the intervention. Attrition bias may also be an issue in these studies since there is unclear bias introduced by the incomplete reporting of outcome data.

The outcomes of the interventions investigated in all of our included studies are summarized in Table 3.

**Table 3:** Effects of interventions on measures of household economic burden

Type of measure and study	Source of data	Measure	Relative difference (95% CI)	*P*	Reference period (months)	Out-of-pocket cost for control group, mean (SD)	Standardized mean difference (95% CI)^a^
**Out-of-pocket costs**							
Choudhry et al. (2011)^31^	Insurance claims	Relative ratio of costs per patient	Prescription drugs: 0.70 (0.65 to 0.75)	< 0.001	12	1164 (1331)	−0.30 (−0.35 to −0.25)
			Non-drug: 0.82 (0.72 to 0.94)	0.005	12	618 (1480)	−0.11 (−0.17 to −0.06)
			All: 0.74 (0.68 to 0.80)	< 0.001	12	1781 (2263)	−0.26 (−0.31 to −0.20)
			Cardiovascular-specific, prescription drugs: 0.49 (0.46 to 0.53)	< 0.001	12	665 (721)	−0.58 (−0.64 to −0.53)
			Cardiovascular-specific, non-drug: 0.91 (0.82 to 1.00)	0.05	12	235 (349)	−0.10 (−0.15 to −0.04)
			Cardiovascular-specific, total: 0.60 (0.56 to 0.64)	0.001	12	900 (888)	−0.50 (−0.55 to −0.45)
Choudhry et al. (2012)^32^	Insurance claims	Relative ratio of costs per patient	Statin, medication and insurance co-payments: 0.05 (NR)^b^	NR	1	11.95 (11.44)	−1.02 (−1.06 to −0.90)
			Statin, medical: 0.90 (0.83 to 0.98)^b^	NR	1	NR	–
			Statin, pharmacy: 0.65 (0.62 to 0.68)^b^	NR	1	NR	–
			Statin, total: 0.79 (0.75 to 0.83)^b^	NR	1	NR	–
			Clopidogrel, medication and insurance co-payments: 0.61 (NR)^b^	NR	1	14.43 (13.38)	−0.43 (−0.50 to −0.35)
			Clopidogrel, medical: 0.76 (0.61 to 0.94)^b^	NR	1	NR	–
			Clopidogrel, pharmacy: 0.72 (0.67 to 0.76)^b^	NR	1	NR	–
			Clopidogrel, total: 0.74 (0.66 to 0.82)^b^	NR	1	NR	–
Davidoff et al. (2005)^33^	Self-report	Change in percentage of patients paying	At least US$ 2000: −1.3 (−8.94 to 6.34)	0.05	12	NR	–
			US$ 500–1999: −4.0 (−14.39 to 6.39)	> 0.05	12	NR	–
			US$ 1–499: 3.2 (−7.19 to 13.59)	> 0.05	12	NR	–
			Nothing: 2.2 (−2.31 to 6.71)	> 0.05	12	NR	–
Goldman et al. (2006)^34^	Insurance claims	Difference in difference change in mean costs per patient of MH/SUD services, US$	National PPO: 4.48 (0.91 to 8.06)	≤ 0.05	24	NR	–
			Mid-Atlantic PPO 1: −15.43 (−26.14 to −4.73)	≤ 0.05	24	NR	–
			Mid-Atlantic PPO 2: −13.82 (−23.96 to −3.67)	≤ 0.05	24	NR	–
			Northeastern PPO 1: −8.78 (−21.14 to 3.57)	> 0.05	24	NR	–
			Northeastern PPO 2: −48·12 (−66.85 to −29.39)	≤ 0.05	24	NR	–
			Western PPO: −49.80 (−61.17 to −38.43)	≤ 0.05	24	NR	–
			Southern PPO: −87.06 (−99.73 to −74.38)	≤ 0.05	24	NR	–
Barry et al. (2013)^29^	Insurance claims	Difference in difference change in mean costs per patient of MH/SUD services, US$	−178 (−257 to −97)^c^	≤ 0.05	12	NR	–
Busch et al. (2013)^30^	Insurance claims	Difference in difference change in mean costs per patient, US$	Bipolar disorder: −148 (−217 to −85)	NR	12	NR	–
			Major depression: −100 (−123 to −77)	NR	12	NR	–
			Adjustment disorder: −68 (−84 to −54)	NR	12	NR	–
Heikkinen et al. (2011)^36^	Self-report	Relative ratio of costs per patient	Total: 0.98 (NR)	NR	NR	240 (264)	−0.02 (−0.35 to 0.32)
			Hospital: 1.04 (NR)	NR	NR	124 (134)	0.04 (−0.30 to 0.37)
			Laboratory tests and X-ray examinations: 0.45 (NR)	NR	NR	216 (242)	−0.64 (−1.29 to 0.01)
			Medication: 1.16 (NR)	NR	NR	26.2 (19.29)	0.17 (−0.20 to 0.53)
			Physician fees: 0.95 (NR)	NR	NR	51.39 (64.24)	−0.05 (−0.58 to 0.48)
			Travel: 1.17 (NR)	NR	NR	18.73 (24.79)	0.11 (−0.36 to 0.57)
			Equipment: 1.10 (NR)	NR	NR	11.63 (8.03)	0.14 (−0.41 to 0.69)
			Medical certificate: 0.83 (NR)	NR	NR	8.02 (9.09)	−0.15 (−0.64 to 0.34)
			Escort: 4.44 (NR)	NR	NR	7.88 (9.19)	0.94 (−0.45 to 2.32)
			Utility bills: 1.71 (NR)	NR	NR	2.49 (1.70)	0.82 (−0.10 to 1.73)
			Parking: 1.27 (NR)	NR	NR	1.49 (1.85)	0.23 (−0.39 to 0.86)
			Additional: 0.29 (NR)	NR	NR	120 (144)	−0.68 (−2.42 to 1.06)
Van Houtven et al. (2013)^35^	Self-report	Logged costs per patient, US$	Caregiver: −54.32 (−143.81 to 35.17)^d^	NR	1	NR	–
			Care-recipient: 192.25 (−361.86 to 746.36)^d^	NR	1	NR	–
			Total: 57.42 (−461.39 to 576.23)^d^	NR	1	NR	–
**Households with catastrophic health expenditure**							
Jing et al. (2013)^37^	Self-report	Difference in difference change in proportion of households, %	0.53 (NR)	> 0.05	12	−2.10 (5.75)	NR
**Delaying the seeking of care because of costs**							
Davidoff et al. (2005)^33^	Self-report	Change in percentage of patients who delayed seeking care due to cost	−1.7 (−6.6 to 3.2)	NR	12	NR	–
**Probability of out-of-pocket costs**							
Van Houtven et al. (2013)^35^	Self-report	Probability that household paid	Any caregiver costs: 0.26 (0.09 to 0.44)	NR	1	NR	–
			Any care-recipient costs: 0.11 (−0.06 to 0.29)	NR	1	NR	–
			Any costs: 0.23 (0.12 to 0.34)	NR	1	NR	–

CI: confidence interval; MH/SUD: mental health and substance use disorders; NR: not reported; PPO: preferred provider organization; SD: standard deviation; US$: United States dollars.

^a^ The difference in mean effects in the intervention and control groups, divided by the pooled standard deviation.

^b^ Relative changes in out-of-pocket costs were adjusted for age, sex, income, race, coronary artery disease, congestive heart failure, diabetes, hypertension, Charlson comorbidity score, number of hospitalizations and prescription drugs on enrolment.

^c^ Difference in difference analysis of gender-, age- and area-adjusted change in annual out-of-pocket costs among those in at least the 90th percentile of MH/SUD treatment expenditure. The corresponding proportion of total MH/SUD costs fell significantly by 5% (*P* ≤ 0.05).

^d^ Confidence interval calculated from reported standard error.

Two studies conducted in the USA evaluated the effectiveness of reducing or eliminating co-payments and found statistically significant reductions in out-of-pocket costs for cardiovascular pharmaceuticals and medical services.^31^^,^^32^ Another three studies conducted in the USA evaluated the effectiveness of parity in service coverage for mental health problems and substance use disorders.^29^^,^^30^^,^^34^ In these three studies, statistically significant reductions in out-of-pocket expenditure were reported for the whole study population,^34^ among children with high expenditure^29^ and in specific disease groups.^30^ For example, the reported mean annual reductions in out-of-pocket costs per patient were 148, United States dollars (US$) for bipolar disease, US$ 100 for major depression and US$ 68 for adjustment disorder.^30^ A sixth study in the USA found a statistically significant association between the expansion of health-insurance coverage and the proportion of people who had moderate out-of-pocket costs of US$ 1–2000 per person.^33^

In rural China, the implementation of a voluntary community-based insurance programme that offered higher reimbursement for outpatient services for a poor population was not found to reduce the prevalence of catastrophic health expenditure significantly.^37^

In Finland, the web-based delivery of information to patients was not associated with any change in out-of-pocket expenditure.^36^ In the USA, an intervention that targeted information at caregivers was found to increase the care-associated spending of the caregivers and had no significant effect on total out-of-pocket expenditure on health for the patients.^35^

Outcomes other than out-of-pocket expenditure were assessed in several studies (Table 4; available from: http://www.who.int/bulletin/volumes/93/2/14-139287). Two insurance interventions were adequately powered to measure their effect on clinical and health-service outcomes. One study found significant reductions in the rates of total major vascular events or revascularization.^31^^,^^32^ None of the other seven studies we included in our systematic review appeared to show a significant impact on the clinical or health-service outcomes assessed – probably because they were underpowered to assess the effect.

**Table 4:** Other patient outcomes assessed in the included studies

Patient outcome	Choudhry et al. (2011)^31^	Choudhry et al. (2012)^32^	Davidoff et al. (2005)^33^	Goldman et al. (2006)^34^	Jing et al. (2013)^37^	Barry et al. (2013)^29^	Busch et al. (2013)^30^	Heikkinen et al. (2011)^36^	Van Houtven et al. (2013)^35^
**Clinical**									
Readmission for major vascular event or coronary revascularization	Yes	No	No	No	No	No	No	No	No
Rate of total major vascular events or revascularization	Yes	Yes	No	No	No	No	No	No	No
**Health-system feature**									
Private health-insurance coverage	No	No	Yes	No	No	No	No	No	No
**Health-service use and access**									
Emergency presentations	No	Yes	No	Yes	No	No	No	No	No
Hospital admissions	No	Yes	No	Yes	No	No	Yes	Yes	No
Physician visits	No	Yes	No	Yes	No	No	Yes	Yes	No
Other^a^	No	No	No	No	No	No	No	Yes	No
Unmet needs^b^	No	No	No	Yes	No	No	No	No	No
**Adherence**									
Medication possession ratio^c^	Yes	No	No	No	No	No	No	No	No
Full adherence	Yes	No	No	No	No	No	No	No	No
Medication filling	No	Yes	No	No	No	No	No	No	No
**Direct and indirect costs**									
Costs to private health insurer	Yes	Yes	No	No	No	No	No	No	No
Time costs^d^	No	No	No	No	No	No	No	Yes	No

^a^ First aid, nurses and other health-care professionals.

^b^ Medical, dental, prescription drugs and mental health services.

^c^ The number of days a patient had a supply of each medication class available divided by the number of days the patient was eligible for that medication.

^d^ Including work and free time spent attending laboratory tests, X-ray examinations and receiving patient-targeted education and time spent off work, on sick leave.

## Discussion

To the authors’ knowledge this is the only systematic review to synthesize published evidence on the effectiveness of interventions that address the diverse ways that illness and injury adversely affect household economics. In the reviewed studies, the economic burden of illness at household level was measured predominantly in terms of out-of-pocket costs. The interventions that were found to be most effective at mitigating the burden of illness were implemented in the context of existing health-insurance schemes and involved reducing or eliminating co-payments for disease-specific treatments. Offering parity in the benefits for specific illnesses also significantly reduced out-of-pocket costs.

However, any reductions in out-of-pocket expenditure should be interpreted in the context of total spending – by the individual and the household – for the management of an illness or injury.^30^ One study reported that, although the 21% reduction in out-of-pocket expenditure found in their study was statistically significant, the absolute annual reduction – of US$ 100–148 per patient – was unlikely to confer protection from catastrophic expenditure.^30^ Total household expenditure on health-related care – including the costs of transport, home assistance, medical equipment and accommodation – can be much greater than the direct out-of-pocket costs of medicines and surgery.^38^ Moreover, such indirect costs of care are seldom covered by health-insurance schemes, particularly in low-income settings. Few of our included studies incorporated other categories of out-of-pocket expenditure beyond the direct costs of medical care. Interventions that solely reduce co-payments for specific aspects of care will only be effective if the care that is covered represents the main economic burden of the illness or injury at household level. Furthermore, many households may have more than one member with illness or injury. Therefore, interventions will need to move beyond targeting disease-specific aspects of treatment and, instead, take a holistic view of the multiple and diverse ways that illness and injury affect household economic circumstances.

Of the nine studies we reviewed, seven involved changes to – or extensions of – an existing package of health-insurance benefits, with the sole aim of shifting the costs of care to the insurer and minimizing the costs to the patient. Only one of these health-insurance studies was conducted in a low- or middle-income country. Although most of the health-insurance interventions were associated with statistically significant effects within the study period, such interventions will not be put into widespread practice unless they can be shown to be economically viable. To the authors’ knowledge, only one of the health-insurance studies was accompanied by a published cost–effectiveness investigation of the type needed to inform priority setting and resource planning for any sustainable intervention. In low- and middle-income countries, the financial sustainability of such measures is critical. If the post-2015 development goals relating to poverty reduction are to be achieved, good evidence is needed to inform the development of stronger and more financially sustainable health systems in these settings.

There is a general scarcity of evaluations of innovative interventions to address the economic burden of illness and injury. Such interventions have the potential to supplement existing health-insurance policies, particularly those being rolled out to achieve universal health coverage in low- and middle-income settings. The interventions uncovered in this review tended to be health-insurance-based or, to a lesser extent, involve some form of patient education. If used in isolation, such interventions cannot resolve the fundamental issues of social disadvantage and poverty and overlook the multidimensional pathways in which illnesses or injuries are linked to economic outcomes. For instance, there appear to have been few attempts to examine the role of strategies such as income support or programmes to support household consumption in addressing the financial challenges of long-term chronic illness. This might be due to the narrow disciplinary perspectives of the relevant researchers.^39^

This review highlights a need for method development in this field, to take account of the capacity of households to afford out-of-pocket expenditure and the impact of coping strategies on household economic outcomes. There is an interconnection and, potentially, a vicious cycle between poor economic circumstances and illness.^3^^,^^40^ Social disadvantages can predispose individuals to a risk of illness. This, in turn, can predispose individuals and their households to illness-related poverty and economic hardship. These economic consequences can further perpetuate poor health, through impaired quality of life, depression and non-adherence to treatment. Interventions to address the economic burden of illness have the potential to break this nexus. However, research has been slow to adopt tools for measuring outcomes in this field beyond out-of-pocket expenditure, and the relevant studies that have been conducted have been of variable quality and rarely randomized controlled trials. There have also been inconsistencies in the measurement and reporting of outcomes such as out-of-pocket costs and catastrophic health expenditures.^41^^,^^42^ Once a consistent approach to measuring outcomes has been developed, research in this area will allow for greater comparability between studies^8^^,^^9^ and offer opportunities for the routine assessment of household expenditures within research on clinical interventions.^43^^,^^44^

This review has limitations. First, the authors of excluded studies were not contacted to determine if they had collected data on relevant outcomes but not reported them. Second, the household economic burden of illness or injury was not the primary outcome in all of the included studies. It is possible that some included studies were not sufficiently powered to detect a change in this outcome. Third, this review was limited to studies published in the peer-reviewed literature. Fourth, most of the included studies were conducted in the USA and so low- and middle-income settings were underrepresented. Finally, there were few randomized controlled trials included. As a result of the two latter issues, our findings are unlikely to be representative of all health systems.

## Conclusion

Health-insurance programmes that reduce or eliminate co-payments for defined illness-specific treatments can effectively provide some financial protection, by reducing out-of-pocket expenditure. However, little is known about the cost–effectiveness of such programmes and about other forms of intervention that may provide relief from adverse economic outcomes to households. Given the multiple and diverse ways that illness and injury can affect the economic circumstances of households, this review highlights the need for method development in this field – above and beyond the limited focus on out-of-pocket expenditure. Additionally, especially in low- and middle-income countries, there is wide scope for research on the effectiveness of innovative non-insurance interventions that could provide low-cost and better-targeted support.
